# Design and Synthesis of Novel 2-Phenylaminopyrimidine (PAP) Derivatives and Their Antiproliferative Effects in Human Chronic Myeloid Leukemia Cells

**DOI:** 10.3390/molecules14104166

**Published:** 2009-10-19

**Authors:** Sheng Chang, Shi-Liang Yin, Jian Wang, Yong-Kui Jing, Jin-Hua Dong

**Affiliations:** 1 Shenyang Pharmaceutical University, Shenyang, 110016, China; E-Mails: changsheng-pharm@hotmail.com (S.C.); yslal@163.com (S-L.Y.); wjmed@163.com (J.W.); 2 Mount Sinai School of Medicine, New York, NY 10029, USA; E-Mail: yongkui.Jing@mssm.edu (Y-K.J.)

**Keywords:** 2-Phenylaminopyrimidine (PAP) derivative, STI-571, antiproliferative activity

## Abstract

A series of novel 2-phenylaminopyrimidine (PAP) derivatives structurally related to STI-571 were designed and synthesized. The abilities of these compounds to inhibit proliferation were tested in human chronic myeloid leukemia K562 cells. (*E*)-3-(2-bromophenyl)-*N*-[4-methyl-3-(4-pyridin-3-yl-pyrimidin-2-ylamino)phenyl]acryla- mide(**12d**) was the most effective cell growth inhibitor and was 3-fold more potent than STI-571.

## Introduction

Chronic myeloid leukemia (CML) is a hematopoietic disorder characterized by the uncontrolled expansion of myeloid progenitors in the bone marrow [1,2,3]. Bcr-Abl tyrosine kinase, a gene product of the reciprocal translocation between chromosomes 9 and 22 has been found to be a main cause of CML and has been used as a successful target for the treatment of CML [4,5,6].

The 2-phenylaminopyrimidine (PAP) derivative STI-571 (4-(4-methylpiperazin-1-ylmethyl)-*N*-[4- methyl-3-(4-pyridine-3-yl-pyrimidin-2-ylamino)phenyl]benzamide mesylate, imatinib mesylate, Gleevec) is a potent and relative selective Bcr-Abl tyrosine kinase inhibitor that has shown potent efficacy in CML patients in chronic phase [7,8]. Although newly diagnosed patients with chronic phase disease achieve durable responses to STI-571 therapy, relapse and resistance are frequently observed in patients with advanced disease [9,10]. Mutations in the kinase domain of Bcr-Abl have been found to be one of the main reasons of resistance to STI-571 [11]. Crystallographic studies have revealed a basic structure of Abl in which mutations in the kinase domain confer STI-571 resistance [12]. STI-571 binds to an inactive conformation of the active loop of Abl. Mutations in the kinase domain influence the residues to which the drug binds or change the conformation of Abl making STI-571 unable to bind [12,13]. Potent Bcr-Abl activity inhibition by 2-phenylaminopyrimidine (PAP) derivatives which bind to Abl in a different way suggests that novel PAP derivatives would be more potent CML cell growth inhibitors than STI-571 [14,15,16,17,18]. Recently, we synthesized a series of novel PAP cinnamamide derivatives structurally related to STI-571 and compared their antiproliferative activity in CML K562 cells. Several derivatives with more potent antiproliferative activity were found.

## Results and Discussion

### Design and synthesis of PAP cinnamamide derivatives

**Figure 1:**
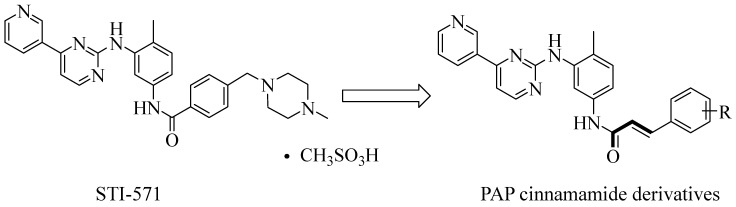
The structures of the designed compounds related to STI-571.

**Scheme 1:**
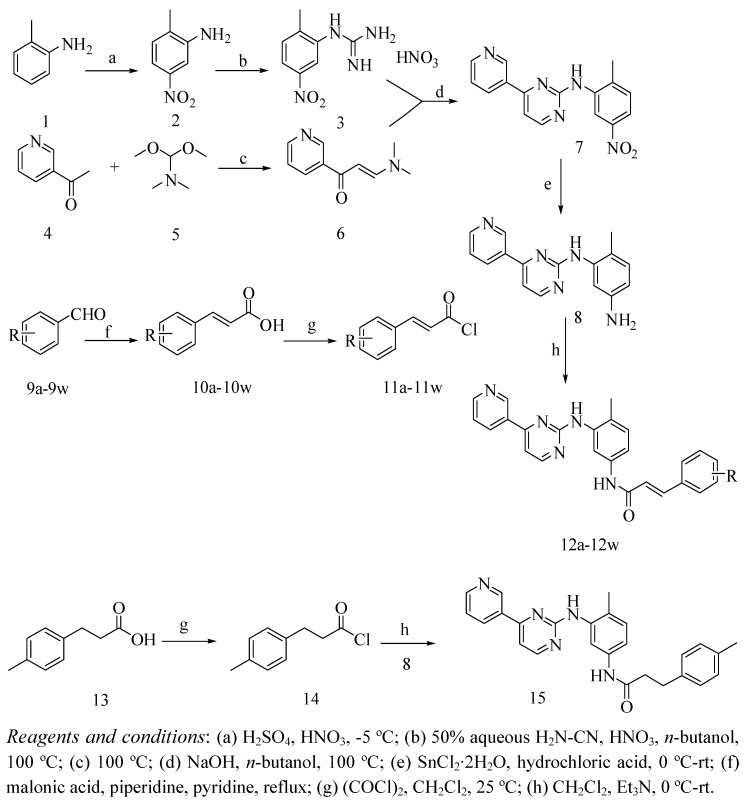
Synthetic routes of the target compounds.

The design of PAP derivatives was based on the vinylogy rule. A double bond was introduced into the benzamide moiety to construct an α,β-unsaturated acylamide (Figure 1), with the reasoning that α,β-unsaturated enones could react with the nuclephilic groups of residues in the Abl kinase domain. Therefore, a series of PAP cinnamamide derivatives with various substituents on the right hand benzene ring were synthesized. In order to investigate the effect of the conjugate moiety, compound **15** was also synthesized. The synthetic routes of the target compounds are shown in Scheme 1.

The general method of Bredereck [14,19] was used to synthesize the pyrimidine ring system, which involved reacting 3-acetylpyridine (**4**) with *N*,*N*-dimethylformamide dimethyl acetal (**5)** to give the enaminone **6** in 92.6% yield. The phenylaminopyrimidine derivative ring system **7** was constructed by reacting enaminone **6** with guanidinium **3**, which was prepared in 66% yield via the reaction of *o*-toluidine **1** with 50% aqueous cyanamide in refluxing *n*-butanol. Reduction of **7** with SnCl_2_·2H_2_O afforded 6-methyl-*N*^1^-(4-pyridin-3-yl-pyrimidin-2-yl)benzene-1,3-diamine (**8**) in 75% yield, which reacted with *trans*-cinnamyl chloride to afford the target compounds **12a-12w** in yields ranging from 33.9% to 78.8%. Compound **15** was synthesized via the same method with 3-*p*-tolylpropanoic acid as the starting material.

### Antiproliferative activities of PAP cinnamamide derivatives in CML cells and proposed binding mode to c-Abl

The antiproliferative activities of these PAP cinnamamide derivatives were tested in human leukemia K562 cells (Table 1), and the structure-activity relationship was analyzed. The results revealed that compounds with sterical small substituents at position 2 or at position 4 on the right hand benzene ring were more potent than the corresponding unsubstituted compound **12a**. Compounds with a substituent at position 2 were more potent than compounds with the same substituent at position 4. Among them, compound **12c** and **12d** were more effective than STI-571 in inhibiting K562 cell growth. Comparison of activities of the compounds with substitution at position 2 revealed that electron-drawing groups were better than alkoxy groups for cell growth inhibition. The order of activity of the compounds with a substituent at position 2 was Br > Cl > NO_2_ > F> OCH_3_ > OCH_2_CH_3_. Introduction of an additional substituent into compound **12f** with a -OCH_3_ at position 3 (i.e. compound **12o**) improved the antiproliferative activity, while other compounds with double substituents were less active than the compounds with a single substituent. Compound **12l** was 7-fold more potent than compound **15**, suggesting that the vinylogy modification might be important for the antiproliferative activity of these PAP cinnamamide derivatives.

The proposed binding mode may partially rationalize above pharmacological results. The most potent compound **12d** was found to share the same binding mode as that of STI-571 observed in the crystal structure of c-Abl kinase. Both STI-571 and compound **12d** form hydrogen bonds with Met318, Thr315, Glu286 and Phe382. Furthermore, the olefinic bond of compound **12d** matches the stereo conformation of the phenyl group of STI-571, which might form π-π interaction with c-Abl residues.

**Figure 2:**
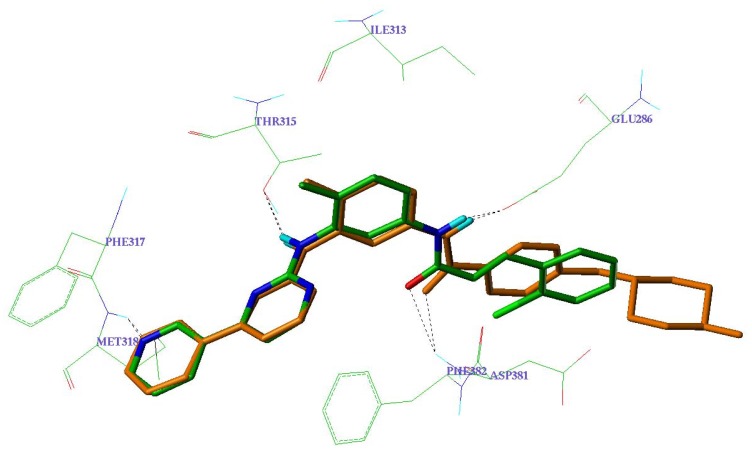
Binding modes of STI-571 (orange; X-ray crystal structure) and compound **12d** (green; docked pose) to the c-Abl kinase domain.

## Experimental

### General

All reagents and solvents (analytical grade) were commercially available and used without further purification. Melting points were determined with a Yanaco apparatus and are uncorrected. ^1^H-NMR spectra were recorded in DMSO-d_6_ (unless otherwise specified) on a Bruker AV-500 spectrometer. The coupling constants were recorded in hertz (Hz) and chemical shifts were reported in parts per million (δ, ppm) downfield from tetramethylsilane (TMS). High-resolution mass spectra (HRMS) were recorded on a high-resonance electrospray time-of-flight mass spectrometer LC/MSD QTOF 6520 (Agilent).

### 2-Methyl-5-nitroaniline *(**2**)*

To sulfuric acid (225 g) cooled at -5 °C, *o*-toluidine (**1**, 15 g, 140 mmol) was added dropwise with vigorous stirring. Mixed acid (14 g of 65% nitric acid and 50 g of sulfuric acid) was then added dropwise at -5 °C during 2 h. Then the mixture was poured onto crushed ice and made alkaline with aqueous sodium hydroxide. The precipitate formed was collected by filtration and air-dried. Subsequent recrystallization from 50% ethanol provided 17.28 g (81%), of product as maroon crystals, mp: 105-107 °C.

### N-(2-Methyl-5-nitrophenyl)guanidinium nitrate *(**3**)*

To a solution of 2-methyl-5-nitroaniline (**2**, 20.46 g, 135 mmol ) in *n*-butanol (120 mL), 65% nitric acid (10.5 mL) was added dropwise followed by a 50% aqueous solution of cyanamide (22.7 mL, 22.81 g, 270 mmol). The reaction mixture was refluxed for 12 h and then cooled to 0 °C. The precipitate was collected by filtration and washed with chilled solution of methanol and diethyl ether (1:1, 20 mL) and air-dried to afford 18.48 g (53%) of the title product as a yellow solid, mp: 216-218 °C. 

### 3-Dimethylamino-1-(pyridin-3-yl)propenone *(**6**)*

A mixture of 3-acetylpyridine (**4**, 24.21 g, 200 mmol) and *N*,*N*-dimethylformamide dimethyl acetal (**5**, 30.96 g, 260 mmol) was refluxed for 16 h under nitrogen and then concentrated under reduced pressure. To the residue, cyclohexane (100 mL) was added, and the mixture was cooled to 0 °C. The precipitate was collected by filtration to afford 32.61 g (92.6%) of product as yellow crystals, mp: 78-80 °C. ^1^H-NMR (CDCl_3_) δ: 9.05 (d, 1H, *J* = 1.6, Py-2-H), 8.68 (dd, 1H, *J*_1_ = 4.8, *J*_2_ = 1.6, Py-6-H), 8.21 (dt, 1H, *J*_1_ = 7.9, *J*_2_ = 1.9, Py-4-H), 7.86 (d, 1H, *J* = 12.2, COCH=CH), 7.37 (dd, 1H, *J*_1_ = 7.3, *J*_2_ = 4.9, Py-5-H), 5.69 (d, 1H, *J* = 12.2, COCH=CH ), 3.20 (s, 3H, CH_3_), 2.97 (s, 3H, CH_3_).

### N-(2-Methyl-5-nitrophenyl)-4-pyridin-3-yl-pyrimidin-2-ylamine *(**7**)*

To a suspension of 3-dimethylamino-1-(pyridin-3-yl)propenone (**6**, 26.96 g, 153 mmol) and *N*-(2-methyl-5-nitrophenyl)guanidinium nitrate (**3**, 51.40 g, 200 mmol) in *n*-butanol (200 mL), solid sodium hydroxide (8.63 g, 216 mmol) was added. The mixture was refluxed for 16 h and then cooled to 0 °C. The precipitate was collected by filtration and washed with methanol and diethyl ether, and air-dried to afford 43.62 g (92.4 %) of product as a yellow solid, mp: 196-197 °C.

### 6-Methyl-N^1^-(4-pyridin-3-yl-pyrimidin-2-yl)benzene-1,3-diamine *(**8**)*

To a solution of stannous chloride dihydrate (11.29 g, 50 mmol) in hydrochloric acid (30 mL) cooled at 0 °C, *N*-(2-methyl-5-nitrophenyl)-4-pyridin-3-yl-pyrimidin-2-ylamine (**7**, 3.69 g, 12 mmol) was added in portions while the suspension was vigorously stirred for 6 h. The mixture was then poured onto crushed ice, made alkaline with solid sodium hydroxide, and extracted three times with ethyl acetate (100 mL). The combined organic phase was dried over anhydrous sodium sulfate and the filtrate was evaporated to dryness *in vacuo*. The residue was recrystallized from methylene chloride to afford 2.52 g (75%) of product as a yellow solid, mp: 142-144 °C. ^1^H-NMR δ: 9.24 (d, 1H, *J* = 2.2, Py-2-H ), 8.69 (dd, 1H, *J*_1_ = 4.8, *J*_2_ = 1.6, Py-6-H), 8.63 (s, 1H, NH), 8.46 (d, 1H, *J* = 5.1, pyrimidyl-6-H), 8.39 (dt, 1H, *J*_1_ = 8.1, *J*_2_ = 2.0, Py-4-H), 7.53 (dd, 1H, *J*_1_ = 7.9, *J*_2_ = 5.1, Py-5-H), 7.35 (d, 1H, *J* = 5.1, pyrimidyl-5-H ), 6.87 (d, 1H, *J* = 8.2), 6.80 (d, 1H, *J* = 2.3), 6.35 (dd, 1H, *J*_1_ = 8.0, *J*_2_ = 2.3), 4.83 (br, 2H, NH_2_), 2.07 (s, 3H, CH_3_).

### General procedure for the synthesis of substituted cinnamic acids ***10a-10w***

Malonic acid (1.92 g 18.33 mmol) and the appropriate substituted benzaldehyde **9a-9w** (12.23 mmol) were dissolved in pyridine (2.5 mL) and a catalytic amount of piperidine was added. The reaction mixture was refluxed for 1-3 h, and then cooled to room temperature and acidified with diluted hydrochloric acid. The precipitate was collected by filtration, air-dried and recrystallized from ethanol to yield corresponding cinnamic acid.

### General procedure for the synthesis of the target compounds ***12a-12w** and **15***

To a solution of cinnamic acid **10a-10w** or **13** (0.84 mmol) in methylene chloride (15 mL), oxalyl chloride (1 mmol) was added. The reaction mixture was stirred for 2-3 h at room temperature, and then evaporated to dryness *in vacuo*. The cinnamoyl chloride or **14** yielded was dissolved in methylene chloride (5 mL) and used in the subsequent reaction. To a solution of 6-methyl-*N*^1^-(4-pyridin-3-yl- pyrimidin-2-yl)benzene-1,3-diamine (**8**, 0.21 g, 0.76 mmol) and triethylamine (0.25g, 2.48 mmol) in methylene chloride (15 mL) cooled at 0 °C, the above methylene chloride solution of the cinnamoyl chloride or **14** was added dropwise and the reaction mixture was then stirred for 2-8 h at room temperature. The precipitate was collected by filtration, washed with methylene chloride and water, and air-dried to afford the corresponding cinnamamide derivatives.

*(E)-N-[4-methyl-3-(4-pyridin-3-yl-pyrimidin-2-ylamino)phenyl]-3-phenylacrylamide* (**12a**): yield: 74.5%; mp:181-182 °C. ^1^H-NMR δ: 10.12 (s, 1H, NHCO), 9.27 (d, 1H, *J* = 1.8, Py-2-H), 8.92 (s, 1H, NH), 8.69 (dd, 1H, *J*_1_ = 4.8, *J*_2_ = 1.6, Py-6-H ), 8.51 (d, 1H, *J* = 5.2, pyrimidinyl-6-H), 8.46 (dt, 1H, *J*_1_ = 8.0, *J*_2_ = 1.8, Py-4-H), 7.99 (d, 1H, *J* = 1.6), 7.61 (d, 2H, *J* = 7.1), 7.57 (d, 1H, *J* = 15.8, COCH=CH), 7.54 (dd, 1H, *J*_1_ = 7.9, *J*_2_ = 4.8, Py-5-H), 7.46-7.39 (m, 5H), 7.19 (d, 1H, *J* = 8.3), 6.84 (d, 1H, *J* = 15.8, COCH=CH), 2.21 (s, 3H, CH_3_); HRMS m/z calcd. for C_25_H_21_N_5_ONa [M+Na]^+^ 430.16438, found: 430.21130.

*(E)-3-(2-fluoropheny)-N-[4-methyl-3-(4-pyridin-3-yl-pyrimidin-2-ylamino)phenyl]acrylamide* (**12b**): yield: 76.8%; mp: 243-245 °C. ^1^H-NMR δ: 10.26 (s, 1H, NHCO), 9.28 (d, 1H, *J* = 1.6, Py-2-H), 8.95 (s, 1H, NH), 8.69 (dd, 1H, *J*_1_ = 4.8, *J*_2_ = 1.6, Py-6-H), 8.52 (d, 1H, *J* = 5.1, pyrimidinyl-6-H), 8.48 (dt, 1H, *J*_1_ = 8.0, *J*_2_ = 1.9, Py-4-H), 8.04 (d, 1H, *J* = 1.9), 7.84 (d, 1H, *J* = 15.6, COCH=CH ), 7.76-7.71 (m, 2H), 7.54 (dd, 1H, *J*_1_ = 7.9, *J*_2_ = 4.8, Py-5-H), 7.50 (t, 1H, *J* = 8.4), 7.44 (d, 2H, *J* = 5.2), 7.37-7.34 (m, 1H), 7.21 (d, 1H, *J* = 8.3), 6.87 (d, 1H, *J* = 15.6, COCH=CH), 2.21 (s, 3H, CH_3_); HRMS m/z calcd. for C_25_H_21_FN_5_O [M+H] ^+^ 426.17301, found: 426.20733.

*(E)-3-(2-chlorophenyl)-N-[4-methyl-3-(4-pyridin-3-yl-pyrimidin-2-ylamino)phenyl]acrylamide* (**12c**): yield: 77.8%; mp: 270-272 °C. ^1^H-NMR δ: 10.24 (s, 1H, NHCO), 9.27 (d, 1H, *J* = 1.8, Py-2-H), 8.92 (s, 1H, NH), 8.69 (dd, 1H, *J*_1_ = 4.8, *J*_2_ = 1.6, Py-6-H), 8.52 (d, 1H, *J* = 5.2, pyrimidinyl-6-H), 8.48 (dt, 1H, *J*_1_ = 8.0, *J*_2_ = 1.9, Py-4-H), 8.01 (d, 1H, *J* = 1.6), 7.87 (d, 1H, *J* = 15.5, COCH=CH), 7.78-7.76 (m, 1H), 7.56-7.51 (m, 2H), 7.45-7.42 (m, 4H), 7.20 (d, 1H, *J* = 8.3), 6.90 (d, 1H, *J* = 15.5, COCH=CH), 2.21 (s, 3H, CH_3_); HRMS m/z calcd. for C_25_H_21_ClN_5_O [M+H] ^+^ 442.14346, found: 442.14943.

*(E)-3-(2-bromophenyl)-N-[4-methyl-3-(4-(pyridin-3-yl-pyrimidin-2-ylamino)phenyl]acrylamide* (**12d**): yield: 80.8%; mp: 268-269 °C. ^1^H-NMR δ: 10.26 (s, 1H, NHCO), 9.28 (d, 1H, *J* = 1.7, Py-2-H), 8.95 (s, 1H, NH), 8.69 (dd, 1H, *J*_1_ = 4.8, *J*_2_ = 1.6, Py-6-H), 8.52 (d, 1H, *J* = 5.1, pyrimidinyl-6-H), 8.49 (dd, 1H, *J*_1_ = 8.0, *J*_2_ = 1.8, Py-4-H), 8.04 (d, 1H, *J* = 1.9), 7.84 (d, 1H, *J* = 15.6, COCH=CH ), 7.76-7.71 (m, 2H), 7.54 (dd, 1H, *J*_1_ = 7.9, *J*_2_ = 4.8, Py-5-H), 7.50 (t, 1H, *J* = 5.2), 7.44 (d, 2H, *J* = 5.2), 7.37-7.34 (m, 1H), 7.21 (d, 1H, *J* = 8.3), 6.87 (d, 1H, *J* = 15.6, COCH=CH), 2.21 (s, 3H, CH_3_); HRMS m/z calcd. for C_25_H_21_BrN_5_O [M+H] ^+^ 486.09295, found: 486.09298.

*(E)-N-[4-methyl-3-(4-(pyridin-3-yl-pyrimidin-2-ylamino)phenyl]-3-(2-nitrophenyl)acrylamid*e (**12e**): yield: 78.8%; mp: 270-272 °C. ^1^H-NMR δ: 10.26 (s, 1H, NHCO), 9.27 (d, 1H, *J* = 1.8, Py-2-H), 8.92 (s, 1H, NH), 8.68 (dd, 1H, *J*_1_ = 4.8, *J*_2_ = 1.6, Py-6-H), 8.52 (d, 1H, *J* = 5.1, pyrimidinyl-6-H), 8.49 (dt, 1H, *J*_1_ = 8.0, *J*_2_ = 1.8, Py-4-H), 8.08 (d, 1H, *J* = 7.9), 8.01 (d, 1H, *J* = 1.8), 7.84 (d, 1H, *J* = 15.8, COCH=CH), 7.82-7.80 (m, 2H), 7.68-7.65 (m, 1H), 7.53 (dd, 1H, *J*_1_ = 8.0, *J*_2_ = 4.8, Py-5-H), 7.44-7.40 (m, 2H), 7.21 (d, 1H, *J* = 8.4), 6.83 (d, 1H, *J* = 15.5, COCH=CH), 2.22 (s, 3H, CH_3_); HRMS m/z calcd. for C_25_H_20_N_6_O_3_Na [M+Na]^+^ 475.14946, found: 475.16374.

*(E)-3-(2-methoxyphenyl)-N-[4-methyl-3-(4-pyridin-3-yl-pyrimidin-2-ylamino)phenyl]acrylamide* (**12f**): yield: 66.3%; mp: 246-247 °C. ^1^H-NMR δ: 10.08 ( s, 1H, NHCO), 9.26 (d, 1H, *J* = 1.7, Py-2-H), 8.92 (s, 1H, NH), 8.69 (dd, 1H, *J*_1_ = 4.8, *J*_2_ = 1.6, Py-6-H ), 8.51 (d, 1H, *J* = 5.2, pyrimidinyl-6-H), 8.46 (dt, 1H, *J*_1_ = 8.0, *J*_2_ = 1.9, Py-4-H), 7.99 (d, 1H, *J* = 1.8), 7.79 (d, 1H, *J* = 15.8, COCH=CH), 7.56 (dd, 1H, *J_1_* = 7.8, *J_2_* = 1.5), 7.53(dd, 1H, *J*_1_ = 8.0, *J*_2_ = 4.8, Py-5-H), 7.43-7.37 (m, 3H), 7.18 (d, 1H, *J* = 8.3), 7.10 (d, 1H, *J* = 8.2), 7.01 (t, 1H, *J* = 7.5), 6.87 (d, 1H, *J* = 15.8, COCH=CH), 3.89 (s, 3H, OCH_3_) 2.21(s, 3H, CH_3_); HRMS m/z calcd. for C_26_H_23_N_5_O_2_Na [M+Na]^+^ 460.17494, found: 460.16676.

*(E)-3-(2-ethoxyphenyl)-N-[4-methyl-3-(4-pyridin-3-yl-pyrimidin-2-ylamino)phenyl]acrylamide* (**12g**): yield: 66.6%; mp: 255-257 °C. ^1^H-NMR δ: 10.09 ( s, 1H, NHCO), 9.26 (d, 1H, *J* = 1.8, Py-2-H), 8.92 ( s,1H, NH), 8.69 (dd, 1H, *J*_1_ = 4.8, *J*_2_ = 1.6, Py-6-H ), 8.51 (d, 1H, *J* = 5.1, pyrimidinyl-6-H), 8.48 (dt, 1H, *J*_1_ = 8.0, *J*_2_ = 1.9, Py-4-H), 8.01 (d, 1H, *J* = 1.6), 7.84 (d, 1H, *J* = 15.8, COCH=CH), 7.57 (dd, 1H, *J*_1_ = 7.8, *J*_2_ = 1.3), 7..53 (dd, 1H, *J*_1_ = 8.0, *J*_2_ = 4.8, Py-5-H), 7.43-7.40 (m, 2H), 7.38-7.35 (m, 1H), 7.18 (d, 1H, *J* = 8.3), 7.08 (d, 1H, *J* = 8.3), 7.01 (t, 1H, *J* = 7.5), 6.85 (d, 1H, *J* = 15.8, COCH=CH), 4.15 (q, 2H, *J* = 7.0, OCH_2_CH_3_).), 2.22 (s, 3H, CH_3_), 1.41(t, 3H, *J* = 7.0, OCH_2_CH_3_); HRMS m/z calcd. for C_27_H_26_N_5_O_2_ [M+H]^+^ 452.20865, found: 452.24296.

*(E)-3-(4-fluorophenyl)-N-[4-methyl-3-(4-pyridin-3-yl-pyrimidin-2-ylamino)phenyl]acrylamide* (**12h**): yield: 70.6%; mp: 194-198 °C. ^1^H-NMR δ: 10.28 (s, 1H, NHCO), 9.26 (d, 1H, *J* = 1.8, Py-2-H), 8.92 (s, 1H, NH), 8.68 (dd, 1H, *J*_1_ = 4.8, *J*_2_ = 1.6, Py-6-H ), 8.51 (d, 1H, *J* = 5.1, pyrimidinyl-6-H), 8.47 (dt, 1H, *J*_1_ = 8.0, *J*_2_ = 1.9, Py-4-H), 7.97(d, 1H, *J* = 1.9), 7.68 (dd, 2H, *J*_1_ = 8.5, *J*_2_ = 5.5), 7.56 (d, 1H, *J* = 15.8, COCH=CH), 7.53 (dd, 1H, *J*_1_ = 8.0, *J*_2_ = 4.8, Py-5-H), 7.43 (d, 1H, *J* = 5.3), 7.40 (d, 1H, *J* = 2.0), 7.28 (t, 2H, *J*_1_ = 8.5), 7.19 (d, 1H, *J* = 8.4), 6.78 (d, 1H, *J* = 15.8, COCH=CH), 2.22 (s, 3H, CH_3_); HRMS m/z calcd. for C_25_H_20_FN_5_ONa [M+Na]^+^ 448.15496, found: 448.13271.

*(E)-3-(4-chlorophenyl)-N-[4-methyl-3-(4-pyridin-3-yl-pyrimidin-2-ylamino)phenyl]acrylamide* (**12i**): yield: 65.8%; mp:268-269 °C. ^1^H-NMR δ: 10.14 (s, 1H, NHCO), 9.26 (d, 1H, *J* = 1.8, Py-2-H), 8.92 (s, 1H, NH), 8.69 (dd, 1H, *J*_1_ = 4.8, *J*_2_ = 1.6, Py-6-H), 8.51 (d, 1H, *J* = 5.2, pyrimidinyl-6-H), 8.46 (dt, 1H, *J*_1_ = 8.0, *J*_2_ = 2.0, Py-4-H), 7.98 (d, 1H, *J* = 1.6), 7.64 (d, 2H, *J* = 8.6), 7.56 (d, 1H, *J* = 15.6, COCH=CH), 7.53 (dd, 1H, *J*_1_ = 8.0, *J*_2_ = 4.8, Py-5-H), 7.50 (t, 2H, *J*_1_ = 8.5), 7.43 (d, 1H, *J* = 5.2), 7.41 (d, 1H, *J* = 1.9), 7.19 (d, 1H, *J* = 8.3), 6.84 (d, 1H, *J* = 15.6, COCH=CH ), 2.21 (s, 3H, CH_3_); HRMS m/z calcd. for C_25_H_21_ClN_5_O [M+H] ^+^ 442.14346, found: 442.14943.

*(E)-3-(4-bromophenyl)-N-[4-methyl-3-(4-pyridin-3-yl-pyrimidin-2-ylamino)phenyl]acrylamide* (**12j**): yield: 49.6%; mp:124-125 °C. ^1^H-NMR δ: 10.14 (s, 1H, NHCO), 9.26 (d, 1H, *J* = 1.8, Py-2-H), 8.92 (s, 1H, NH), 8.69 (dd, 1H, *J*_1_ = 4.8, *J*_2_ = 1.6, Py-6-H), 8.52 (d, 1H, *J* = 5.1, pyrimidinyl-6-H), 8.46 (dt, 1H, *J*_1_ = 8.0, *J*_2_ = 1.9, Py-4-H), 7.98 (d, 1H, *J* = 1.5), 7.64 (d, 2H, *J* = 8.5), 7.59 (d, 1H, *J* = 15.6, COCH=CH), 7.53 (dd, 1H, *J*_1_ = 8.0, *J*_2_ = 4.8, Py-5-H), 7.50 (t, 2H, *J*_1_ = 8.5), 7.43 (d, 1H, *J* = 5.2), 7.41 (d, 1H, *J* = 1.9), 7.19 (d, 1H, *J* = 8.3), 6.86 (d, 1H, *J* = 15.7, COCH=CH ), 2.21 (s, 3H, CH_3_); HRMS m/z calcd. for C_25_H_20_BrN_5_ONa [M+Na]^+^ 508.07489, found: 508.05714.

*(E)-N-[4-methyl-3-(4-pyridin-3-yl-pyrimidin-2-ylamino)phenyl]-3-(4-nitrophenyl)acrylamide* (**12k**): yield: 70.0%; mp: 252-254 °C. ^1^H-NMR δ: 10.28 (s, 1H, NHCO), 9.27 (d, 1H, *J* = 1.8, Py-2-H), 8.94 (s, 1H, NH), 8.69 (dd, 1H, *J*_1_ = 4.8, *J*_2_ = 1.7, Py-6-H), 8.52 (d, 1H, *J* = 5.2, pyrimidinyl-6-H), 8.47 (dt, 1H, *J*_1_ = 8.0 Hz, *J*_2_ = 1.9, Py-4-H), 8.29-8.27 (m, 2H), 8.00 (d, 1H, *J* = 1.9) 7.89 (d, 2H, *J* = 8.9), 7.67 (d, 1H, *J* = 15.8, COCH=CH), 7.53 (dd, 1H, *J*_1_ = 8.0, *J*_2_ = 4.8, Py-5-H), 7.44-7.42 (m, 2H), 7.21 (d,1H, *J* = 8.4), 7.02 (d, 1H, *J* = 15.8, COCH=CH), 2.22 (s, 3H, CH_3_); HRMS m/z calcd. for C_25_H_20_N_6_O_3_Na [M+Na]^+^ 475.14946, found: 475.13600.

*(E)-N-[4-methyl-3-(4-pyridin-3-yl-pyrimidin-2-ylamino)phenyl]-3-p-tolylacrylamide* (**12l**): yield: 68.8%; mp: 205-208 °C. ^1^H-NMR δ: 10.07 (s, 1H, NHCO), 9.26 (d, 1H, *J* = 1.8, Py-2-H), 8.92 (s, 1H, NH), 8.68 (dd, 1H, *J*_1_ = 4.8, *J*_2_ = 1.6, Py-6-H), 8.51 (d, 1H, *J* = 5.1, pyrimidinyl-6-H), 8.47 (dt, 1H, *J*_1_ = 8.1, *J*_2_ = 1.9, Py-4-H), 7.97 (d, 1H, *J* = 1.9), 7.53 (dd, 1H, *J*_1_ = 8.0, *J*_2_ = 4.8, Py-5-H), 7.52 (d, 1H, *J* = 15.6, COCH=CH), 7.50 (d, 2H, *J* = 8.0), 7.42 (d, 1H, *J* = 5.2), 7.40 (d, 1H, *J* = 2.0), 7.25 (d, 2H, *J* = 8.0), 7.18 (d, 1H, *J* = 8.4), 6.78 (d, 1H, *J* = 15.7, COCH=CH), 2.33 (s, 3H, CH_3_), 2.22 (s, 3H, CH_3_); HRMS m/z calcd. for C_25_H_24_N_5_O [M+H]^+^ 442.19809, found: 422.19907

*(E)-3-(4-methoxyphenyl)-N-[4-methyl-3-(4-pyridin-3-yl-pyrimidin-2-ylamino)phenyl]acrylamide* (**12m**): yield: 63.3%; mp: 115-117 °C. ^1^H-NMR δ: 10.03 (s, 1H, NHCO), 9.27 (d, 1H, *J* = 1.8, Py-2-H), 8.92 (s,1 H, NH), 8.69 (dd, 1H, *J*_1_ = 4.8, *J*_2_ = 1.6, Py-6-H ), 8.51 (d, 1H, *J* = 5.1, pyrimidinyl-6-H), 8.46 (dt, 1H, *J*_1_ = 8.0, *J*_2_ = 1.9, 1H, Py-4-H), 7.97 (d, 1H, *J* = 1.7), 7.56 (d, 2H, *J* = 8.8), 7.53 (dd, 1H, *J*_1_ = 8.0, *J*_2_ = 4.8, Py-5-H), 7.52 (d, 1H, *J* = 15.6, COCH=CH), 7.42 (d, 1H, *J* = 5.2), 7.40 (d, 1H, *J* = 2.0), 7.18 (d, 1H, *J* = 8.4), 7.01 (d, 2H, *J* = 8.8), 6.69 (d, 1H, *J* = 15.7, COCH=CH), 3.80 (s, 3H, OCH_3_), 2.21 (s, 3H, CH_3_); HRMS m/z calcd. for C_26_H_24_N_5_O_2_ [M+H]^+^ 438.19300, found: 438.17750. 

*(E)-3-(4-benzyloxyphenyl)-N-[4-methyl-3-(4-pyridin-3-yl-pyrimidin-2-ylamino)phenyl]acrylamide* (**12n**): yield: 51.4%; mp: 221-222 °C. ^1^H-NMR δ: 10.02 (s, 1H, NHCO), 9.26 (d, 1H, *J* = 1.8, Py-2-H), 8.91 (s, 1H, NH), 8.69 (dd, 1H, *J*_1_ = 4.8, *J*_2_ = 1.6, Py-6-H ), 8.49 (d, 1H, *J* = 5.1, pyrimidinyl-6-H), 8.45 (dt, 1H, *J*_1_ = 8.1, *J*_2_ = 1.9, Py-4-H), 7.97 (d, 1H, *J* = 1.7), 7.56 (d, 2H, *J* = 8.7), 7.53 (dd, 1H, *J*_1_ = 8.0, *J*_2_ = 4.8, Py-5-H), 7.51 (d, 1H, *J* = 15.7, COCH=CH), 7.45 (d, 2H, *J* = 7.3), 7.43-7.39 (m, 4H), 7.35-7.32 (m, 1H), 7.18 (d, 1H, *J* = 8.4), 7.08 (d, 2H, *J* = 8.7), 6.70 (d, 1H, *J* = 15.6, COCH=CH), 5.20 (s, 2H, OCH_2_Ph), 2.20 (s, 3H, CH_3_); HRMS m/z calcd. for C_32_H_28_N_5_O_2_ [M+H]^+^ 514.22430, found: 514.23057.

*(E)-3-(2,3-dimethoxyphenyl)-N-[4-methyl-3-(4-pyridin-3-yl-pyrimidin-2-ylamino)phenyl]acrylamide* (**12o**): yield: 33.9%; mp: 146-147 °C. ^1^H-NMR δ: 10.13 (s, 1H, NHCO), 9.27 (d, 1H, *J* = 1.8, Py-2-H ), 8.91(s, 1H, NH), 8.69 (dd, 1H, *J*_1_ = 4.8, *J*_2_ = 1.6, Py-6-H ), 8.52 (d, 1H, *J* = 5.1, pyrimidinyl-6-H), 8.46 (dt, 1H, *J*_1_ = 8.0, *J*_2_ = 1.9, Py-4-H), 8.00 (d, 1H, *J* = 1.5), 7.76 (d, 1H, *J* = 15.8, COCH=CH), 7.53 (dd, 1H, *J*_1_ = 8.0, *J*_2_ = 4.8, Py-5-H), 7.43 (d, 1H, *J* = 5.1), 7.41 (d, 1H, *J* = 1.9), 7.20-7.18 (m, 2H), 7.14 (t, 1H, *J* = 8.0), 7.10 (dd, 1H, *J_1_* = 8.1, *J_2_* = 1.7), 6.87 (d, 1H, *J* = 15.8, COCH=CH ), 3.84 (s, 3H, OCH_3_), 3.78 (s, 3H, OCH_3_*),* 2.21 (s, 3H, CH_3_); HRMS m/z calcd. for C_27_H_25_N_5_O_3_Na [M+Na]^+^ 490.18551, found: 490.19200.

*(E)-3-(2,4-dimethoxyphenyl)-N-[4-methyl-3-(4-pyridin-3-yl-pyrimidin-2-ylamino)phenyl]acrylamide* (**12p**): yield: 65.1%; mp:154-156 °C. ^1^H-NMR δ: 9.98 (s, 1H, NHCO), 9.26 (d, 1H, *J* = 1.8, Py-2-H), 8.91(s, 1H, NH), 8.69 (dd, 1H, *J*_1_ = 4.8, *J*_2_ = 1.6, Py-6-H), 8.51 (d, 1H, *J* = 5.1, pyrimidinyl-6-H), 8.46 (dt, 1H, *J*_1_ = 8.1, *J*_2_ = 1.9, Py-4-H), 7.97 (d, 1H, *J* = 1.9), 7.70 (d, 1H, *J* = 15.7, COCH=CH), 7.53 (dd, 1H, *J*_1_ = 8.0, *J*_2_ = 4.8, Py-5-H), 7.50 (d, 1H, *J* = 8.5), 7.42 (d, 1H, *J* = 5.1), 7.40 (d, 1H, *J* = 1.9), 7.17 (d, 1H, *J* = 8.4), 6.74 (d, 1H, *J* = 15.7, COCH=CH), 6.61-6.60 (m, 2H), 3.88 (s, 3H, OCH_3_), 3.82 (s, 3H, OCH_3_), 2.21 (s, 3H, CH_3_); HRMS m/z calcd. for C_27_H_25_N_5_O_3_Na [M+Na]^+^ 490.18551, found: 490.19263.

*(E)-N-[4-methyl-3-(4-pyridin-3-yl-pyrimidin-2-ylamino)phenyl]-3-(2,3,4–trimethoxyphenyl) acrylamide* (**12q**): yield: 69.0%; mp:226-227 °C. ^1^H-NMR δ: 10.05 (s, 1H, NHC=O), 9.26 (d, 1H, *J* = 1.8, Py-2-H), 8.91(s, 1H, NH), 8.69 (dd, 1H, *J*_1_ = 4.8, *J*_2_ = 1.6, Py-6-H), 8.51(d, 1H, *J* = 5.1, pyrimidinyl-6-H), 8.48 (dt, 1H, *J*_1_ = 8.1, *J*_2_ = 1.9, Py-4-H), 7.99 (d, 1H, *J* = 1.9), 7.65 (d, 1H, *J* = 15.8, COCH=CH), 7.53 (dd, 1H, *J*_1_ = 8.0, *J*_2_ = 4.8, Py-5-H), 7.43 (d, 1H, *J* = 5.1), 7.40 (d, 1H, *J* = 1.9), 7.33 (d, *J* = 8.8), 7.18 (d, 1H, *J* = 8.4), 6.91( d, 1H, *J* = 8.8), 6.79 (d, 1H, *J* = 15.8, COCH=CH), 3.84 (s, 3H, OCH_3_), 3.83 (s, 3H, OCH_3_), 3.78 (s, 3H, OCH_3_), 2.21 (s, 3H, CH_3_); HRMS m/z calcd. for C_28_H_27_N_5_O_4_Na [M+Na]^+^ 520.19607, Found: 520.19694.

*(E)-3-(3,4-dimethoxyphenyl)-N-[4-methyl-3-(4-pyridin-3-yl-pyrimidin-2-ylamino)phenyl]acrylamide* (**12r**): yield: 67.8%; mp: 234-235 °C. ^1^H-NMR δ: 10.03 (s, 1H, NHCO), 9.27 (d, 1H, *J* = 1.8, Py-2-H), 8.92 (s,1H, NH), 8.69 (dd, 1H, *J*_1_ = 4.8, *J*_2_ = 1.6, Py-6-H ), 8.50 (d, 1H, *J* = 5.1, pyrimidinyl-6-H), 8.46 (dt, 1H, *J*_1_ = 8.1, *J*_2_ = 1.9, Py-4-H), 7.98 (d, 1H, *J* = 1.9), 7.53 (dd, 1H, *J*_1_ = 8.0, *J*_2_ = 4.8, Py-5-H), 7.50 (d, 1H, *J* = 15.8, COCH=CH), 7.42-7.40 (m, 2H), 7.21 (d, 1H, *J* = 1.8), 7.18 (dd, 2H, *J*_1_ = 8.6, *J*_2_ = 3.1), 7.01 (d, 1H, *J* = 8.4), 6.71(d, 1H, *J* = 15.8, COCH=CH), 3.82 (s, 3H, OCH_3_), 3.80 (s, 3H, OCH_3_)_,_ 2.21 (s, 3H, CH_3_); HRMS m/z calcd. for C_27_H_26_N_5_O_3_ [M+H]^+^ 468.20357, found: 468.20356.

*(E)-3-(4-ethoxy-3-methoxyphenyl)-N-[4-methyl-3-(4-pyridin-3-yl-pyrimidin-2-ylamino]phenyl]acryla-mide* (**12s**): yield: 68.8%; mp: 256-258 °C. ^1^H-NMR δ: 10.03 (s, 1H, NHCO), 9.26 (d, 1H, *J* = 1.8, Py-2-H), 8.92 (s, 1H, NH), 8.68 (dd, 1H, *J*_1_ = 4.8, *J*_2_ = 1.6, Py-6-H), 8.51(d, 1H, *J* = 5.2, pyrimidinyl-6-H), 8.47 (dt, 1H, *J*_1_ = 8.1, *J*_2_ = 1.9 Py-4-H), 7.97 (d, 1H, *J* = 1.9), 7.53 (dd, 1H, *J*_1_ = 8.0, *J*_2_ = 4.8, Py-5-H), 7.50 (d, 1H, *J* = 15.6,COCH=CH), 7.42 (d,1H, *J* = 5.1), 7.40 (d, 1H, *J* = 2.0), 7.20 (d, 1H, *J* = 1.9), 7.18 (d, 1H, *J* = 8.4), 7.15 (dd, 1H, *J_1_* = 8.4, *J_2_* = 1.9), 6.99 (d, 1H, *J* = 8.4), 6.70 (d, 1H, *J* = 15.7, COCH=CH ), 4.05 (q, 2H, *J* = 7.0, OCH_2_CH_3_), 3.81 (s, 3H, OCH_3_), 2.22 (s, 3H, CH_3_), 1.34 (t, 3H, *J* = 7.0, OCH_2_CH_3_); HRMS m/z calcd. for C_28_H_28_N_5_O_3_ [M+H]^+^ 482.21921, found: 482.22009.

*(E)-3-(3-ethoxy-4-methoxyphenyl)-N-[4-methyl-3-(4-pyridin-3-yl-pyrimidin-2-ylamino)phenyl]acryla-mide* (**12t**): yield: 68.5%; mp: 263-264 °C. ^1^H-NMR δ: 10.01 (s, 1H, NHC=O), 9.26 (d, 1H, *J* = 1.8, Py-2-H), 8.92 (s, 1H, NH), 8.68 (dd, 1H, *J*_1_ = 4.8, *J*_2_ = 1.6, Py-6-H), 8.51 (d, 1H, *J* = 5.2, pyrimidinyl -6-H), 8.47 (dt, 1H, *J*_1_ = 8.1, *J*_2_ = 1.9, Py-4-H), 7.97 (d, 1H, *J* = 1.9), 7.53 (dd, 1H, *J*_1_ = 8.0, *J*_2_ = 4.8, Py-5-H), 7.50 (d, 1H, *J* = 15.6, COCH=CH)), 7.42 (d,1H, *J* = 5.1), 7.40 (d, 1H, *J* = 2.1), 7.19-7.16 (m, 3H), 7.00 (d,1H, *J* = 8.4), 6.70 (d, 1H, *J* = 15.7, COCH=CH), 4.06(q, 2H, *J* = 6.9, OCH_2_CH_3_) 3.80 (s, 3H, OCH_3_), 2.21 (s, 3H, CH_3_), 1.35 (t, 3H, *J* = 6.9, OCH_2_CH_3_); HRMS m/z calcd. for C_28_H_28_N_5_O_3_ [M+H]^+^ 482.21921, Found: 482.22036.

*(E)-3-(4-benzyloxy-3-methoxyphenyl)-N-[4-methyl-3-(4-pyridin-3-yl-pyrimidin-2-ylamino)phenyl] acrylamide* (**12u**): yield: 36.4%; mp: 178-179 °C. ^1^H-NMR δ: 10.08 ( s, 1H, NHCO), 9.27 (d, 1H *J* = 1.8, Py-2-H), 8.98 (s, 1H, NH), 8.69 (dd, 1H, *J*_1_= 4.8, *J*_2_ = 1.6, Py-6-H ), 8.51 (d, 1H, *J* = 5.1, pyrimidinyl-6-H), 8.46 (dt, 1H, *J*_1_ = 8.1, *J*_2_ = 1.9, Py-4-H), 7.97 (d, 1H, *J* = 1.7), 7.53 (dd, 1H, *J*_1_ = 8.0, *J*_2_ = 4.8, Py-5-H), 7.51 (1H, *J* = 15.7, COCH=CH), 7.49-7.47 (m, 2H), 7.44-7.40 (m, 4H), 7.36-7.33 (m, 1H), 7.33 (d, 1H, *J* = 1.6), 7.22 (dd, 1H, *J_1_* = 8.4, *J_2_* = 1.8), 7.18 (d, 1H, *J* = 8.4), 7.05 (d, 1H, *J* = 8.7), 6.70 (d, 1H, *J* = 15.6, COCH=CH), 5.20 (s, 2H, OCH_2_Ph), 3.83 (s, 3H, OCH_3_), 2.20 (s, 3H, CH_3_);HRMS m/z calcd. for C_33_H_30_N_5_O_3_ [M+H]^+^ 544.23486, found: 544.23565.

*(E)-3-(3-benzyloxy-4-methoxyphenyl)-N-[4-methyl-3-(4-pyridin-3-yl-pyrimidin-2-ylamino)-phenyl] acrylamide* (1**2v**) yield: 42.7%; mp: 232-234 °C. ^1^H-NMR δ: 10.04 ( s, 1H, NHCO), 9.27(d, 1H, *J* = 1.8, Py-2-H), 8.92( s,1 H, NH), 8.69 (dd, 1H, *J*_1_ = 4.8, *J*_2_ = 1.6, Py-6-H ), 8.51 (d, 1H, *J* = 5.1, pyrimidinyl-6-H), 8.46 (dt, 1H, *J*_1_ = 8.1, *J*_2_ = 1.9, Py-4-H), 7.97 (d, 1H, *J* = 1.7), 7.53 (dd, 1H, *J*_1_ = 8.0, *J*_2_ = 4.8, Py-5-H), 7.50 (d, 1H, *J* = 15.8, COCH=CH), 7.46-7.44 (m, 2H), 7.42-7.38 (m, 4H), 7.35-7.33 (m, 1H), 7.23 (d, 1H, *J* = 1.9), 7.18 (d, 1H, *J* = 8.4), 7.15 (dd, 1H, *J*_1_ = 8.4, *J*_2_ = 1.8), 7.09 (d, 1H, *J* = 8.4), 6.70 (d, 1H, *J* = 15.6, COCH=CH), 5.13 (s, 2H, OCH_2_Ph), 3.83 (s, 3H, OCH_3_), 2.20 (s, 3H, CH_3_); HRMS m/z calcd. for C_33_H_30_N_5_O_3_ [M+H]^+^ 544.23486, found: 544.23565.

*(E)-3-benzo[1,3]dioxol-5-yl-N-[4-methyl-3-(4-pyridin-3-yl-pyrimidin-2-ylamino)phenyl]acrylamide* (**12w**): yield: 46.8%; mp: 244-246 °C. ^1^H-NMR δ: 10.02 (s, 1H, NHCO), 9.26 (d, 1H, *J* = 1.2, Py-2-H), 8.91 (s, 1H, NH), 8.69 (dd, 1H, *J*_1_ = 4.5, *J*_2_ = 1.1, Py-6-H), 8.51 (d, 1H, *J* =5.1, pyrimidinyl-6-H), 8.46 (dt, 1H, *J*_1_ = 8.1, *J*_2_ = 1.9, Py-4-H), 7.97 (d, 1H, *J* = 1.3), 7.53 (dd, 1H, *J*_1_ = 8.0, *J*_2_ = 4.8, Py-5-H), 7.49 (d, 1H, *J*=15.6, COCH=CH), 7.42 (d, 1H, *J* = 5.1), 7.40 (d, 1H, *J* = 1.6), 7.18 (d, 2H, *J* = 7.2), 7.13 (dd, 1H, *J*_1_ = 8.1, *J*_2_ = 1.4), 6.97 (d, 1H, *J* = 8.0), 6.67 (d, 1H, *J* = 15.6, COCH=CH), 6.08 (s, 2H, OCH_2_O), 2.21 (s, 3H, CH_3_); HRMS m/z calcd. for C_26_H_21_N_5_O_3_Na [M+Na]^+^ 474.15421, found: 474.14769.

*N-[4-methyl-3-(4-pyridin-3-yl-pyrimidin-2-ylamino)phenyl]-3-p-tolylpropionamide* (**15**): yield: 76.6%; mp: 168-169°C. ^1^H-NMR δ: 9.81(s, 1H, NHCO), 9.26 (d, 1H, *J* = 1.8, Py-2-H), 8.90 (s, 1H, NH), 8.69 (dd, 1H, *J*_1_ = 4.7, *J*_2_ = 1.6, Py-6-H ), 8.50 (d, 1H, *J* = 5.1, pyrimidinyl-6-H), 8.46 (dt, 1H, *J*_1_ = 8.0, *J*_2_ = 1.8, Py-4-H), 7.88 (d, 1H, *J* = 1.6), 7.51 (dd, 1H, *J*_1_ = 8.0 Hz, *J*_2_ = 4.8, Py-4-H), 7.41 (d, 1H, *J* = 5.1), 7.28 (dd, 1H, *J*_1_ = 8.0, *J*_2_ = 1.8), 7.14-7.12 (m, 3H), 7.07 (d, 2H, *J* = 8.0), 2.86 (t, 2H, *J =* 7.45, COCH_2_CH_2_), 2.58 (t, 2H, *J =* 7.45, COCH_2_CH_2_), 2.24 (s, 3H, CH_3_), 2.19 (s, 3H, CH_3_); HRMS m/z calcd. for C_26_H_26_N_5_O [M+H]^+^ 424.21374, found: 424.24776.

### Antiproliferative activity assay

Human chronic myeloid leukemia K562 cells containing Bcl-Abl fusion kinase were cultured in RPMI 1640 medium (Gibco; New York, NY, USA) containing 10% fetal bovine serum. Cells were seeded at 4.0 × 10^4^ cells/mL and incubated with various concentrations of test compounds for 72 h. Total cell number in each group was counted with the aid of a hemocytometer. The growth inhibition ability was calculated and expressed as the ratio of the cell number in treated group to that in the untreated group. The concentration that inhibited half of the cell growth (IG_50_) was calculated [20].

### Molecular modeling

Molecular docking was carried out using Molegro Virtual Docker (MVD) [21]. The small molecules and the X-ray crystal structure of c-Abl in complex with STI-571 (PDB code: 1iep[12]) were imported, and STI-571 was used to define the binding cavity. Molecular docking was carried out using the Molegro Virtual Docker (MVD) [21]. The docking algorithm was set at 1,500 maximum iterations with a simplex evolution population size of 50 and a minimum of 10 runs. The schematic diagrams of interactions between c-Abl and docked poses were analyzed by Sybyl 6.91 package [22].

## Conclusions

A series of novel PAP cinnamamide derivatives structurally related to STI-571 were synthesized and evaluated for their antiproliferative activity toward human leukemia K562 cells. Compounds **12c** and 1**2d** with a substitution of Cl or Br at position 2 were the most effective compounds in inhibiting K562 cell growth, being 2-3 fold more potent than STI-571. The effects of these compounds on the inhibition of Bcr-Abl activity are worthy of further study.

## Figures and Tables

**Table 1:** The substituents of target compounds and their antiproliferative effects in K562 cells.

Compound	R	GI_50_ (μM) in K562^a^
**12a**	H	2.01 ± 0.10
**12b**	2-F	0.67 ± 0.07
**12c**	2-Cl	0.10 ± 0.01
**12d**	2-Br	0.07 ± 0.01
**12e**	2-NO_2_	0.26 ± 0.02
**12f**	2-OCH_3_	0.83 ± 0.05
**12g**	2-OCH_2_CH_3_	1.55 ± 0.21
**12h**	4-F	1.83 ± 0.09
**12i**	4-Cl	1.26 ± 0.04
**12j**	4-Br	0.55 ± 0.05
**12k**	4-NO_2_	0.39 ± 0.04
**12l**	4-CH_3_	0.51 ± 0.02
**12m**	4-OCH_3_	1.05 ± 0.06
**12n**	4-OCH_2_Ph	> 5
**12o**	2,3-di-OCH_3_	0.14 ± 0.01
**12p**	2,4-di-OCH_3_	1.26 ± 0.05
**12q**	2,3,4-tri-OCH_3_	0.78 ± 0.06
**12r**	3,4-di-OCH_3_	0.99 ± 0.17
**12s**	3-OCH_3_,4-OCH_2_CH_3_	> 5
**12t**	3-OCH_2_CH_3_,4-OCH_3_	0.48 ± 0.04
**12u**	3-OCH_3_,4-OCH_2_Ph	> 5
**12v**	3-OCH_2_Ph,4-OCH_3_	> 5
**12w**	3,4(-OCH_2_O-)	0.57 ± 0.02
**15**		3.51 ± 0.33
**STI-571**		0.23 ± 0.01

^a^ GI_50_ is the concentration that inhibits 50% of cell growth. Cells were seeded at 4.0 × 10^4^ cells/mL and incubated with various concentrations of tested compounds for 72 h. The data shown are the mean ± SE of three independent experiments.
